# Supplementary material for: VAS3947 Induces UPR-Mediated Apoptosis through Cysteine Thiol Alkylation in AML Cell Lines
Source: Int J Mol Sci. 2020 Jul 31;21(15):5470. doi: 10.3390/ijms21155470 (PMC7432790; doi:10.3390/ijms21155470)
Supplement: Supplementary file 1 [file ijms-21-05470-s001.pdf]

Supplementary material

Supplementary materials and methods.

Hydrogen peroxide (H_2O_2) incubation

Cells were seeded in a 6 well plate at 2 x 10^5 cells/mL and preincubated for 1 h w/wo hydrogen peroxide (H_2O_2; 100 µM). Then, VAS3947 (4 µM) or DMSO was added and incubated at 37°C. After 72 h, alive cells were manually enumerated by Trypan blue exclusion, using a microscope.

Supplementary figures

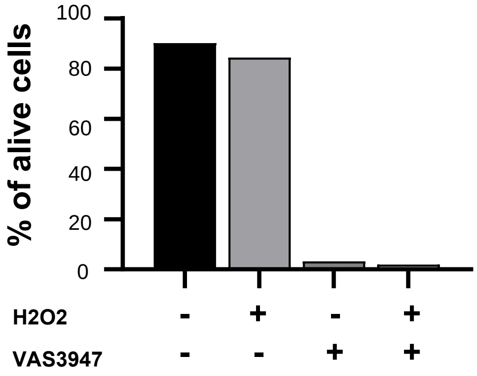

Figure S1. Effect of H_2O_2 (100 µM) supplementation on MV-4-11 cells incubated with VAS3947 at 4 µM (*n* = 1).

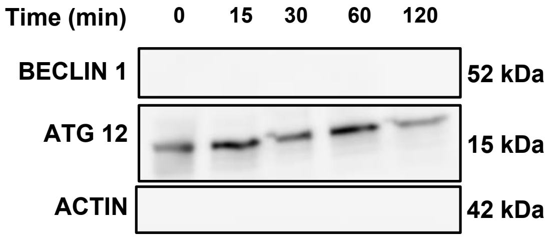

Figure S2. Effect of VAS3947 (4 µM) on the expression of autophagy proteins in MV-4-11 cells.
